# Combined treatment with ultrasound-targeted microbubble destruction technique and NM-aFGF-loaded PEG-nanoliposomes protects against diabetic cardiomyopathy-induced oxidative stress by activating the AKT/GSK-3β1/Nrf-2 pathway

**DOI:** 10.1080/10717544.2020.1785052

**Published:** 2020-07-02

**Authors:** Ming Zhang, Ning-Wei Zhu, Wei-Cheng Ma, Meng-Jia Chen, Lei Zheng

**Affiliations:** a Department of Pharmacy, Ningbo Yinzhou NO.2 Hospital, Ningbo, China; b Department of Pharmacy, Zhejiang Pharmaceutical College, Ningbo, China; c Department of Ultrasonography, Henan Provincial People's Hospital, Zhengzhou University People's Hospital, Zhengzhou, China; d Department of Ultrasonography, the First Affiliated Hospital of Wenzhou Medical University, Wenzhou, China

**Keywords:** Diabetic cardiomyopathy, NM-aFGF, UTMD, oxidative stress

## Abstract

The present study sought to investigate the effect of non-mitogenic acidic fibroblast growth factor (NM-aFGF) loaded PEGylated nanoliposomes (NM-aFGF-PEG-lips) combined with the ultrasound-targeted microbubble destruction (UTMD) technique on modulating diabetic cardiomyopathy (DCM)and the mechanism involved. Animal studies showed that the diabetes mellitus (DM) group exhibited typical myocardial structural and functional changes of DCM. The indexes from the transthoracic echocardiography showed that the left ventricular function in the NM-aFGF-PEG-lips + UTMD group was significantly improved compared with the DM group. Histopathological observation further confirmed that the cardiomyocyte structural abnormalities and mitochondria ultrastructural changes were also significantly improved in the NM-aFGF-PEG-lips + UTMD group compared with DM group. The cardiac volume fraction (CVF) and apoptosis index in the NM-aFGF-PEG-lips + UTMD group decreased to 10.31 ± 0.76% and 2.16 ± 0.34, respectively, compared with those in the DM group (CVF = 21.4 ± 2.32, apoptosis index = 11.51 ± 1.24%). Moreover, we also found significantly increased superoxide dismutase (SOD) activity and glutathione peroxidase (GSH-Px) activity as well as clearly decreased lipid hydroperoxide levels and malondialdehyde (MDA) activity in the NM-aFGF-PEG-lips + UTMD group compared with those in the DM group (*p* < .05). Western blot analysis further revealed the highest level of NM-aFGF, p-AKT, p-GSK-3β1, Nrf-2, SOD2 and NQO1 in the NM-aFGF-PEG-lips + UTMD group. This study confirmed using PEGylated nanoliposomes combined with the UTMD technique can effectively deliver NM-aFGF to the cardiac tissue of diabetic rats. The NM-aFGF can then inhibit myocardial oxidative stress damage due to DM by activating the AKT/GSK/Nrf-2 signaling pathway, which ultimately improved the myocardial structural and functional lesions in diabetic rats.

## Introduction

Diabetic cardiomyopathy (DCM) represents one of the major cardiovascular complications in diabetic patients and manifests with impaired diastolic and systolic cardiac function that may lead to heart failure (Galderisi., 2006; Pappachan et al., 2013); however, there remains no effective treatment. Given that oxidative stress and DNA damage have been considered to be the critical mechanisms for the development of DCM (Giacco & Brownlee, 2010), attenuation of diabetes-induced oxidative damage and the subsequent cardiac apoptosis and fibrosis are expected to exert beneficial effects and may be a potential novel therapeutic strategy for DCM.

Fibroblast growth factor (FGF) is a super family of heparin-binding growth factors including at least 23 members. As the considered prototype member of the growth factor family, acidic fibroblast growth factor (aFGF or FGF1) is a multipotent factor associated with cell proliferation, differentiation and survival during development by initiating various intracellular signal transduction pathways (Chen M et al., 2020). It is reported that aFGF is highly expressed in the heart and participates in heart development (Xiao et al., 2010; Guan et al., 2016). There is also evidence of the survival promotion of aFGF due to its antiapoptotic and vasoactive properties in oxidative stress-induced damage and ischemia and reperfusion injuries in heart (Tian et al., 2017). In addition, animal research has suggested that aFGF effectively reduces blood glucose and reverses insulin resistance in insulin resistant and type 2 diabetes mice (Scarlett et al., 2016). aFGF is thus a potentially valuable therapeutic agent for DCM treatment. However, aFGF has powerful mitogenic and proliferative influences, producing potential tumourigenic ability (Pili et al., 1997).

Non-mitogenic aFGF (NM-aFGF) was generated by genetic engineering, and it has lost only the potential to stimulate cell proliferation with all other functions compared with aFGF (Chen et al., 2009). NM-aFGF has great potential in the treatment of DCM. However, the current administration methods for the delivery of exogenous NM-aFGF or NM-aFGF genes to myocardial tissue include direct cardiac injection or myocardial administration, which are inconvenient and highly risky in clinical application. There is a strong need to establish an efficient and safe mode of NM-aFGF delivery for the treatment of DCM.

Nano-liposomes are useful as efficient delivery vehicles for drugs, plasmids, peptides and proteins as they have high solubility and bioavailability and low toxicity and are non-immunogenic (Yuba et al., 2013; Aisha et al., 2014). PEGylated nanoliposomes obtained by incorporating a polyethylene glycol phospholipid (DSPE-PEG2000) into stable liposomes can protect the liposomes from being recognized and cleared by the reticuloendothelial systems (Allen & Cullis, 2013; Bozzuto & Molinari, 2015). The use of PEGylated nanoliposomes as the carrier of NM-aFGF may improve the stability of NM-aFGF both during storage and in blood circulation and greatly increase the drug circulation time. However, additional strategies to increase the selectivity of NM-aFGF for cardiac tissue are still needed and will improve the drug delivery to the heart without causing unnecessary impact on the other body tissues.

Ultrasound-targeted microbubble destruction (UTMD) is a useful tool to promote the specific delivery of therapeutics to some organs. Several studies have shown that UTMD can obviously improve the organ specificity of *in vivo* drug release from liposomes (Sheng et al., 2018). After intravenous injection, microbubbles can arrive at the myocardial tissue and be destroyed by an ultrasound beam, and the resultant cavitation effect is able to enhance the release of drug from liposome in the ultrasonic sites (Miller & Dou, 2009; Panje et al., 2012; Lentacker et al., 2014). unlike focused therapeutic ultrasound, the low-intensity ultrasound generated by diagnostic devices can induce cavitation effects with less significant thermal effects than high intensity applications. Meanwhile, micro-streaming and radiation forces have been found to have minor effects for low-intensity ultrasound exposure (Ahmadi et al., 2012; Cui et al., 2020). The damage of UTMD against the focused tissues was negligible at the low intensity. The UTMD technique therefore holds considerable promise as an effective strategy to achieve targeted delivery of NM-aFGF loaded nano-liposomes to the myocardium.

**Figure 1.**
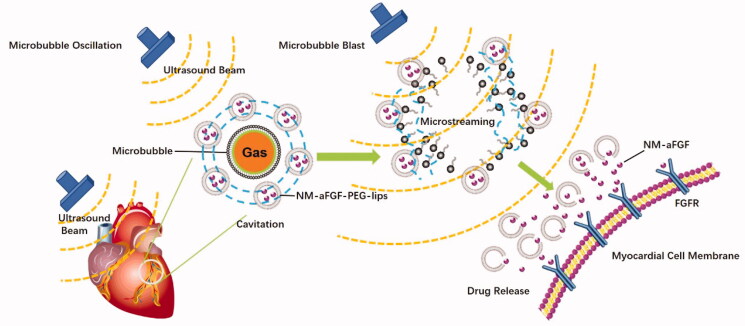
Schematic of NM-aFGF-PEG-lips combined with UTMD mediated NM-aFGF for DCM therapy.

In this study, we developed new NM-aFGF-loaded PEGylated nano-liposomes (NM-aFGF-PEG-lips) combination therapy with the UTMD technique for simultaneous loading and targeted delivery of NM-aFGF to diabetic hearts. As illustrated in Figure 1, when exposed to the low intensity ultrasound, microbubbles would lead to a cavitation (the oscillations of microbubbles). Such oscillations created a liquid flow around the microbubbles, the so-called microstreaming. When these oscillating microbubbles were blasted by the ultrasound beam, the microstreaming would generate a shear stress on the adjacent nanoliposomes. The UTMD induced elevated shear stress levels can enhance the release of drug from nanoliposomes in myocardial cells. To test whether applying this innovative formulation for drug delivery is effective in preventing DCM, the STZ-induced type 1 diabetic animal model was established, and a broad range of commonly used pathophysiological indicators of heart conditions were measured. These measurements allowed thorough preclinical evaluation of the *in vivo* effects of 16 weeks of NM-aFGF-PEG-lips + UTMD treatment on cardiac functions and related structural damage. We found that chronic administration of this combination therapy can indeed protect against diabetes-induced cardiac dysfunction and may be developed as an effective strategy to prevent DCM in future clinical therapy.

## Methods

### Preparation and characterization of NM-aFGF-PEG-lips

#### Preparation of PEG modified NM-aFGF nano-liposomes

The PEG modified NM-aFGF nano-liposomes were prepared by the water-in-water emulsion and freeze-drying technique as described previously (Zhao et al., 2014). In brief, 2 mL of 2.0% w/v homogeneous gelatin solution containing a high concentration NM-aFGF was emulsified in 1 mL of 20% w/v poloxamer 188 solution of 0.1% w/v D, L-glyceraldehyde by sonication (110 w, 20 s) at 37 °C using a probe sonicator (Scientz Biotechnology Co. Ltd., China). The emulsion was then bathed at 5 °C under moderate magnetic stirring at 2500 rpm to form modified nanoparticles. After 5 h of stirring, the emulsion was lyophilized to gain NM-aFGF-loaded gelatin nanoparticle powder. Then, lyophilized NM-aFGF-loaded gelatin nanoparticle powder was dispersed in a solution containing DSPE-PEG2000, trehalose and cholesterol. By sonication (90 w, 20 s) at 25 °C, the mixture suspension was then lyophilized to yield lyophilized powder containing NM-aFGF-PEG-lips, which was reconstituted in double-distilled water to form a suspension for administration. Blank liposomes (blank-lips) were prepared using gelation solution instead of NM-aFGF gelatin solution.

#### Characterization of NM-aFGF-PEG-lips

The morphology of NM-aFGF-PEG-lips was analyzed by scanning electron microscopy (SEM). The zeta potential and size of the NM-aFGF-PEG-lips were measured in Malvern ZetaSize (Malvern Instruments Ltd., India). Approximately 1 mL each of the NM-aFGF-PEG-lips and blank-lips were assigned to the zeta cell, and measurements were recorded. The experiment was performed at 25 °C.

The encapsulation efficiency of NM-aFGF-PEG-lips was determined as previously described (Zhao et al., 2014). The ultracentrifugation method was used to separate free NM-aFGF from liposomes (Sheng et al., 2018). First, 1.5 mL of the NM-aFGF-PEG-lips dispersion was centrifuged at 10,000 g for 40 min. The supernatant was then collected and diluted for NM-aFGF determination using an ELISA kit. The encapsulation efficiency was calculated as indicated below. The analyses were performed in triplicate.

$$\text{Encapsulation}\;\text{efficiency}\left(\%\right)=\frac{\left(\text{total}\;\text{amount}\;\text{of}\;\text{drug}\;–\;\text{amount}\;\text{of}\;\text{drug}\;\text{in}\;\text{supernatant}\right)}{\text{total}\;\text{amount}\;\text{of}\;\text{drug}}\times 100\%$$

### Preparation and suspension of phospholipid-based microbubbles

According to our previous study, phospholipid-based microbubbles (PMB) were prepared by sonication lyophilization (Lu et al., 2011). In brief, hydrogenated phosphatidylcholine (>99% purity, Doosan Corporation Biotech BU, Korea), polyethylene glycol 1500 (Qingming Chemical Plant, China), and poloxamer 188 (Shenyang Chemical Plant, China) were dissolved in n-butanol (analytical grade, Beijing Chemical Plant, China) and sonicated at 30 °C using a JY 92-II ultrasonic processor (KunShan US Instrument Inc., China) at a frequency of 40 kHz and power of 160 W for 3 min. The PMB solution was frozen at −20 °C and lyophilized at 5 × 10 − 4 Pa pressure for 20 h (at −48 °C for 15 h, then warmed up to 10 °C < 5 h). For experiments, PMB solution was prepared by reconstituting one vial of lyophilized PMB (150 mg) with 2 mL 0.9% w/v NaCl solution. The PMB concentration in the solution formed was approximately 2 × 10^9^ bubble/mL with an average diameter of 3.4 μm as measured by a Coulter counter (Coulter Corporation, Hialeah, FL).

### Animals studies

#### Establishment of a type 1 diabetes mellitus model

Male Sprague-Dawley (SD) rats, approximately 200 g, were purchased from Shanghai Slake Laboratory Animal Co., Ltd. The animals were handled according to protocols approved by the ethics committee of Wenzhou Medical University, and all animal-handling procedures were performed according to the Guide for the Care and Use of Laboratory Animals from the National Institutes of Health.

After one week of acclimatization prior to the experimental procedures, diabetes mellitus (DM) was induced in the rats by intraperitoneal single injection of STZ (70 mg/kg, Sigma-Aldrich; St. Louis, MO, USA). On the 3rd and 7th days and at 2 weeks after STZ administration, fasting blood was collected from the tail vein, and samples were analyzed for blood glucose using a glucometer (Aque-Check, Roche, Basel, Switzerland). Animals with fasting blood glucose levels greater than 250 mg/dL that stabilized over next two weeks were considered diabetic. The rats in the control group were injected with citrate buffer without STZ.

#### Experimental group and administration methods

The DM rats were then assigned randomly and equally (ten rats per group) to five groups, including the DM group (DM rats were treated with 1 mL saline), NM-aFGF group (DM rats were treated with 20 ug/kg NM-aFGF in 1 mL saline), NM-aFGF-PEG-lips group (DM rats were treated with 20 ug/kg NM-aFGF-PEG-lips in 1 mL saline), NM-aFGF + UTMD group (DM rats were treated with a mixture of 20 ug/kg NM-aFGF and PMB in 1 mL saline combined with ultrasound treatment), and NM-aFGF-PEG-lips + UTMD group (DM rats were treated with a mixture of 20 ug/kg NM-aFGF-PEG-lips and PMB in 1 mL saline combined with ultrasound treatment). NM-aFGF and PMB were administered via the tail vein, and the control group received 1 mL saline injection. All treatments were administered three times a week on Monday, Wednesday and Friday for 16 consecutive weeks. The body weight and blood glucose of the rats in each group were detected before and after drug intervention.

The UTMD delivery procedure was performed as we previously reported (Zhang et al., 2016). The animals were anesthetized with 2% isoflurane before the experiment. The thoracic region was shaved, and the rats were placed in supine position. The linear array transducer (15L8-w probe, 12–14 MHz) was placed over the heart of the rat (depth = 3.0–4.0 cm) to view the papillary muscles of the ventricle. The experimental solution was infused into the tail vein of the sedated rats. When a large number of PMBs were seen entering the heart, the ultrasound pulses were produced to blast the PMBs (MI = 1.9, exposure time =10 s, repeat three times with off-intervals of 1 s to allow refilling of the tissue with more microbubbles). After the bursting, the microbubbles completely disappeared from the heart.

#### Echocardiography

After drug treatment, all animals underwent transthoracic echocardiography to detect cardiac function. The rats were lightly anesthetized with inhaled isoflurane-o_2_ and imaged in recumbent position. Two-dimensional and M-mode recording were used to measure the following cardiac structures: left ventricle end-diastolic diameter (LVEDD), left ventricle end-systolic diameter (LVESD), left ventricle end-diastolic volume (LVEDV), left ventricle end-systolic volume (LVESV), fractional shortening (FS), and ejection fraction (EF). All derived measures by echocardiography were obtained by averaging the readings of three consecutive beats.

#### Tissue preparation and HE staining

After the completion of cardiac function, the rats were sacrificed, and their hearts were arrested in diastole by intraventricular injection of potassium chloride solution (10%w/v). One part of the left ventricular tissue samples from the papillary muscle level was obtained and stored in 2.5% glutaraldehyde for electron microscopes studies. Another part of the samples was embedded in paraffin and cut into 5 μm serial paraffin sections. The remaining tissues were cryopreserved in liquid nitrogen for molecular analyses, and 5 μm slices of heart tissue were stained with hematoxylin and eosin (HE). Morphological examination was performed using a light microscope.

#### Masson staining

After deparaffinization and rehydration, the 5 μm cross-sections were stained with Masson staining and photographed by a polarized microscope (Olympus, America) connected to a video camera (Nikon, Japan). Magnification was set up at ×200. Perivascular fibrosis was not assessed. The collagen volume fraction (CVF) was calculated using Image Pro Plus 6.0 as the percentage of red-stained area in the section.

#### Transmission electron microscopy

The left ventricular samples stored in 2.5% glutaraldehyde were cut into approximately 1 mm^3^ pieces for electron microscopy. The myocardial tissues were then fixed in 1% osmic acid. After dehydration by acetone and embedded in Epon 812, the tissues were sectioned at 1 μm and stained with toluidine blue. Ultrathin sections were cut from blocks and mounted on copper grids. The grids were then counterstained with uranyl acetate and lead citrate solutions, examined in a JEM-1230 (JEOL, Tokyo, Japan) transmission electron microscope.

### Measurement of cardiac lipid hydroperoxide level and MDA, SOD and GSH-px levels

The lipid hydroperoxide level, superoxide dismutase (SOD), glutathione peroxidase (GSH-px) and malondialdehyde (MDA) levels in the heart tissue were estimated using commercially available lipid hydroperoxide, SOD, GSH-px and MDA assay kits obtained from the Nanjing Jiangcheng Bioengineering Institute (Nanjing, Jiangsu, China) following the instructions of the manufacturer. The data were analyzed spectrophotometrically with a SpectraMax M5 instrument (Molecular Devices, CA, USA).

#### Immunohistochemical staining

Immunohistochemistry staining was used to detect the accumulation of collagen I, collagen III, TGF-β11 and caspase-3. The sections of each group were incubated in 3% hydrogen peroxide (H_2_O_2_) for blocking endogenous peroxidase activity and incubated with primary antibody against collagen I (1:50), collagen III (1:50), TGFβ1 (1:1000) and cleaved caspase-3 (1:500) at 4 °C overnight. The sections were then incubated with corresponding secondary antibodies and labeled with horse radish peroxidase after being washed with PBS for 3 min × 5 times. Staining was achieved by DAB after being washed with PBS and counterstained with hematoxylin. The slides were washed briefly, mounted on resinene, and observed under a light microscope. The staining intensity was analyzed using image-pro Plus 6.0 software.

#### Western blotting assay

The Western blotting protocol was described in our previous study (Zhao et al., 2016). The total protein of the heart tissue was isolated from homogenized tissues with TRIzol reagent (Invitrogen, Carlsbad, CA) using standard Invitrogen protocols. After quantifying with bicinchoninic acid (BCA), the proteins were separated by 10% sodium dodecyl sulfate-polyacrylamide gel electrophoresis (SDS-PAGE) and transferred to a polyvinylidene fluoride (PVDF) membrane. After blocking with 5% skim milk, the membrane was incubated overnight in primary antibody (FGF1, AKT, p-AKT, GSK-3α/β, p-GSK-3β, Nrf2, SOD2, NQO1, 1:1000, Abcam). The membranes were then washed with TBST and incubated with specific horseradish peroxidase-conjugated secondary antibody. Quantity One software was used to analyze the blots after detection using enhanced chemiluminescence system.

#### TUNEL staining

TUNEL assay was used to assess the cell apoptosis. The 5 μm sections were deparaffinized by washing in a graded ethanol series. According to the instructions of the manufacturer, DNA fragments were determined using an ApopTag in situ apoptosis detection kit (Biochemicals, Roche, CH). The optical microscope was used to quantify the TUNEL positive cells at 400 magnification. The apoptosis index was measured by counting the number of positive apoptotic cells under 20 visual high-power fields and presented as the mean number of apoptotic cells per high-power field.

### Statistical analysis

One-way ANOVA and Student’s t-test or Kruskal-Wallis test were adopted for statistical comparison using the SAS 8.01 (1999–2000, SAS Institute Inc., Cary, NC, USA). The difference was considered to be statistically significant when the *p*-value was equal or less than .05.

## Results

### Characterization of blank and NM-aFGF-PEG-lips

**Figure 2.**
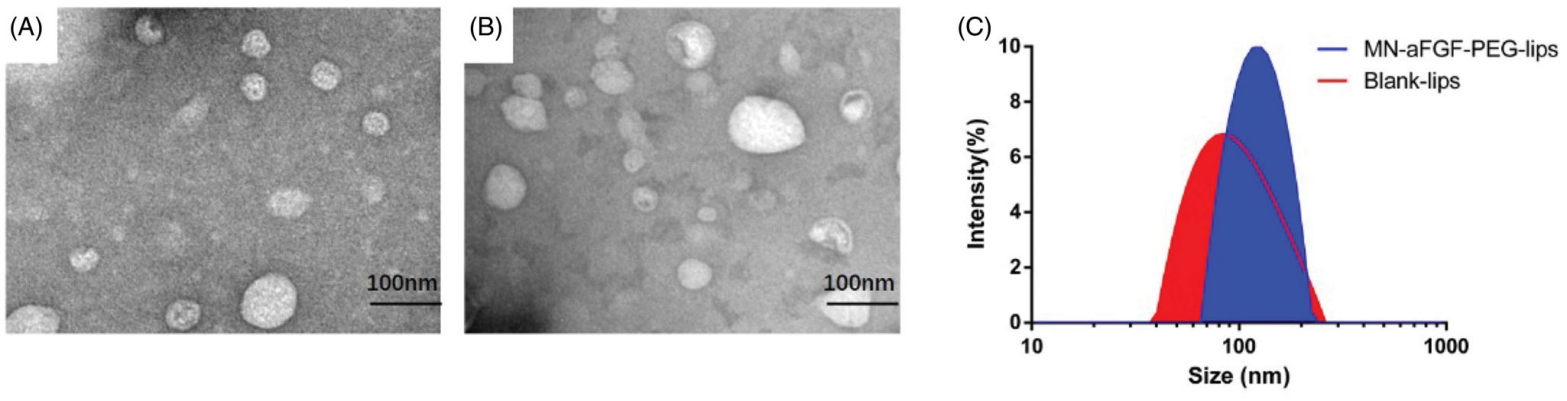
Characterization of blank and NM-aFGF-PEG-lips. (A) SEM of the blank liposomes. (B) SEM of the NM-aFGF-PEG-lips. (C) Size distribution of the blank and NM-aFGF-PEG-lips. NM-aFGF-PEG-lips: non-mitogenic acidic fibroblast growth factor; PEGylated: liposomes; SEM: scanning electron microscopy.

The blank liposomes and NM-aFGF-PEG-lips are clearly depicted in Figure 2(A,B). The blank and NM-aFGF encapsulated liposomes were uniformly distributed and generally spherical in shape with a diameter of nearly 100 nm. There was no significant change in the morphology of the liposomes before and after drug loading. In addition, the dynamic light scattering results revealed that the average particle size of the blank and NM-aFGF-PEG-lips was 83.97 ± 1.31 nm and 125 ± 2.14 nm, respectively (Figure 2(C)). The zeta potential measurements were extremely significant for the functional performance of the nanoliposomes (Huang et al., 2019). The zeta potential values of the blank and NM-aFGF loaded liposomes were −32.3 ± 1.3 and −23.7 ± 1.1, respectively, suggesting that the liposomes may be stable with minimal aggregation. Furthermore, the encapsulation efficiency of NM-aFGF into liposomes was 87.9 ± 2.1%, indicating that the NM-aFGF could be effectively encapsulated by PEG-liposomes.

### NM-aFGF-PEG-lips combined with UTMD treatment against myocardial injury in DM rats

**Figure 3.**
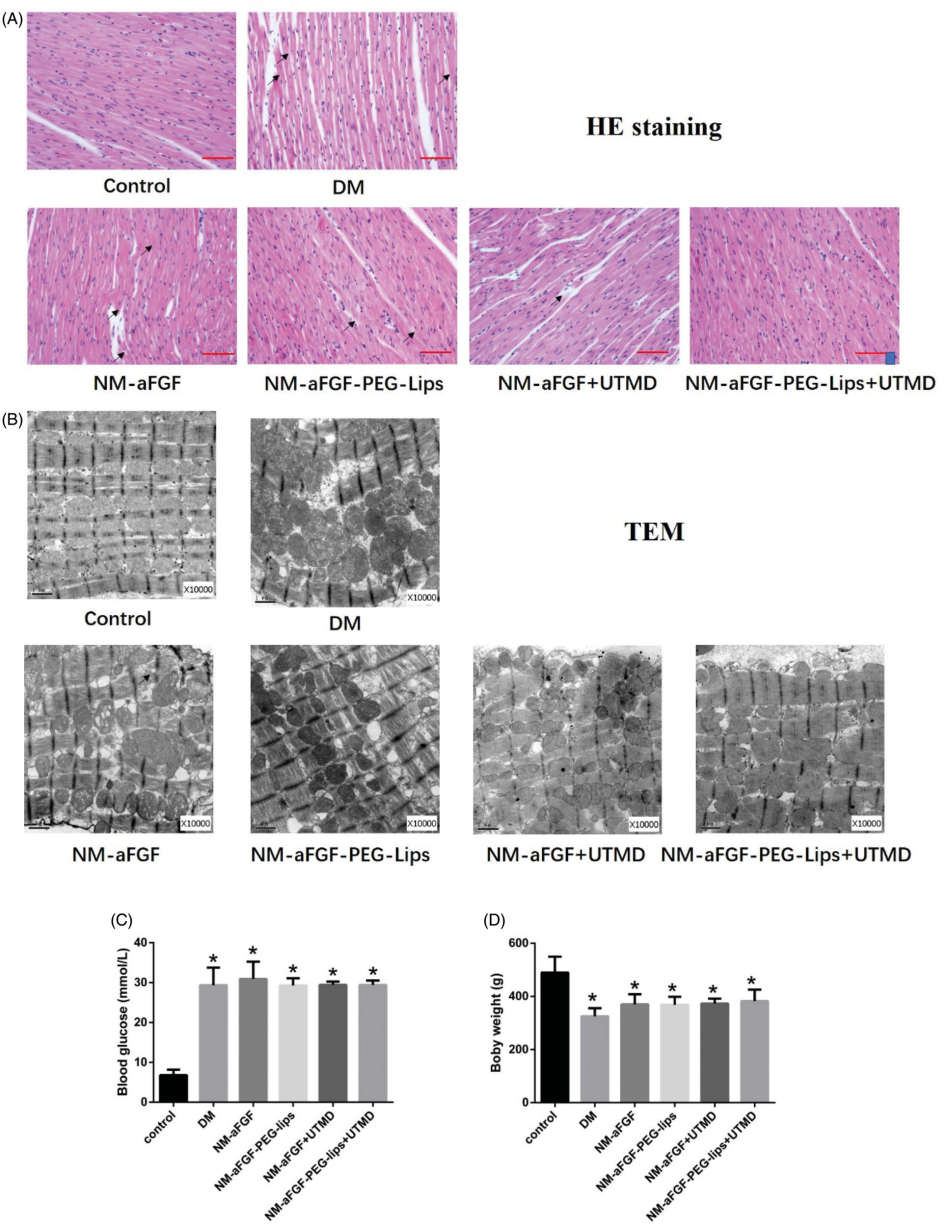
Effect of NM-aFGF-PEG-lips combine with UTMD treatment against myocardial injury. (A) Paraffin-embedded heart tissue was stained with hematoxylin and eosin and examined under microscope (400 × ,bar = 50μm). Arrows indicate cytoplasmic vacuolization, cardiomyocyte necrosis, myofibrillar loss and degeneration. (B) Representative transmission electron micrographs of cardiac tissues. (C) Quantitative analysis of the body weight of rats. *N* = 10 per group. (D) Quantitative analysis of the blood glucose of rats. *N* = 10 per group. *Data are presented as Mean ± SD. ^a^p < .05 vs normal control group.* NM-aFGF-PEG-lips: non-mitogenic acidic fibroblast growth factor- PEGylated -liposomes; DM: diabetes mellitus; UTMD: ultrasound-targeted MB destruction; TEM: transmission electron micrographs.

Myocardial injury was evaluated by HE staining. The body weight and blood glucose were also measured. In the control group, the myocardial structural was clear and well organized, without degeneration and destruction in the myofibrils (Figure 3(A)). In contrast, perinuclear vacuolization, necrosis and myofibrillary degeneration were obvious in the DM group (Figure 3(A)). However, fewer alterations in myocardial structural were observed in the NM-aFGF-treated groups compared with the DM group (Figure 3(A)). Moreover, myocardial structural abnormalities such as necrosis were rare, and vacuolization and myofibrillar loss were not detected under the microscope in the NM-aFGF-PEG-lips + UTMD group. The transmission electron micrographs (TEM) further verified the protective effect of NM-aFGF-PEG-lips combined with UTMD treatment against myocardial injury in a more microscopic view (Figure 3(B)). The ultrastructure of the cardiomyocytes from rat hearts of the control group displayed a normal myocardial structure in which regular sarcomeres comprised continuous myofibrils with numerous mitochondria distributed in an orderly manner between myofibrils. In the cardiomyocytes of the DM group, the myofibrils were disrupted and irregular, and the mitochondria were enlarged and rounded with the cristae appearing disordered. However, these irregular and disordered structures were all dramatically improved by NM-aFGF treatment. Among all NM-aFGF treatment groups, the damage to myocardial ultrastructure in the NM-aFGF-PEG-lips + UTMD-treated rats was markedly attenuated compared with the other NM-aFGF-treated groups.

Body weight and blood glucose are regarded as general indicators of diabetes. Compared with control group, the body weight was significantly decreased (Figure 3(C)) in the DM group (*p* < .05). The blood glucose in the DM group was significantly elevated (Figure 3(D)) compared with that in the control group (*p* < .05). There was no significant difference in body weight or blood glucose between the DM group and all forms of NM-aFGF-treated groups.

### NM-aFGF-PEG-lips reverse cardiac function alterations in diabetic rats

The cardiac structural evaluation was performed by echocardiogram after drug treatment. Through echocardiography, there was a significant decrease in left ventricular ejection fraction (LVEF) and fractional shortening (FS) in the DM group compared with the normal control group (Table 1). After all forms of aFGF treatment, the values of LVEF and FS were significantly increased (*p* < .05); moreover, the NM-aFGF-PEG-lips + UTMD group had the most pronounced increase (*p* < .05). Other parameters of cardiac function such as left ventricle end-diastolic diameter (LVEDD), left ventricle end-systolic diameter (LVESD), left ventricle end-diastolic volume (LVEDV) and left ventricle end-systolic volume (LVESV) were also detected. The DM group exhibited a significant increase in LVEDD, LVESD, LVEDV and LVESV compared with the control group (*p* < .05). After the drug treatment, the values of LVEDD, LVESD, LVEDV and LVESV were significantly decreased compared with the DM group; in addition, the NM-aFGF-PEG-lips + UTMD group had the most pronounced decrease (*p* < .05).

**Table 1.** Results of LVEDD, LVESD, LVEDV, LVESV, LVEF and LVFS in experimental animals (*n* = 10).

Group	LVEDD (mm)	LVESD (mm)	LVEDV (mL)	LVESV (mL)	LVEF (%)	LVFS (%)
Control group	6.45 ± 0.25	3.50 ± 0.16	0.618 ± 0.067	0.110 ± 0.015	82.15 ± 2.32	45.67 ± 2.55
DM group	7.85 ± 0.27^a^	5.18 ± 0.26^a^	1.067 ± 0.102^a^	0.336 ± 0.049^a^	68.65 ± 2.35^a^	34.06 ± 1.74^a^
NM-aFGF	7.47 ± 0.29^bc^	4.75 ± 0.30^bc^	0.929 ± 0.102^bc^	0.263 ± 0.048^bc^	71.82 ± 2.97^bc^	36.46 ± 2.39^bc^
NM-aFGF-PEG-lips	7.17 ± 0.31^bc^	4.35 ± 0.27^bc^	0.830 ± 0.098^bc^	0.206 ± 0.035^bc^	75.23 ± 3.28^bc^	39.25 ± 2.83^bc^
NM-aFGF + UTMD	7.48 ± 0.23^bc^	4.73 ± 0.14^bc^	0.927 ± 0.070^bc^	0.262 ± 0.019^bc^	71.61 ± 3.41^bc^	36.60 ± 3.01^bc^
NM-aFGF-PEG-lips + UTMD	6.83 ± 0.28^b^	3.89 ± 0.19^b^	0.726 ± 0.083^b^	0.148 ± 0.021^b^	79.46 ± 2.61^b^	43.02 ± 2.55^b^

Data are presented as Mean ± SD. ^a^*p* < .05 vs control group; ^b^*p* < .05 vs DM group; ^c^*p* < .05 vs NM-aFGF-PEG-lips + UTMD. NM-aFGF-PEG-lips: non-mitogenic acidic fibroblast growth factor- PEGylated -liposomes; DM: diabetes mellitus; UTMD: ultrasound-targeted MB destruction.

### Effects of NM-aFGF-PEG-lips combined with UTMD on cardiac interstitial fibrosis of diabetic rats

**Figure 4.**
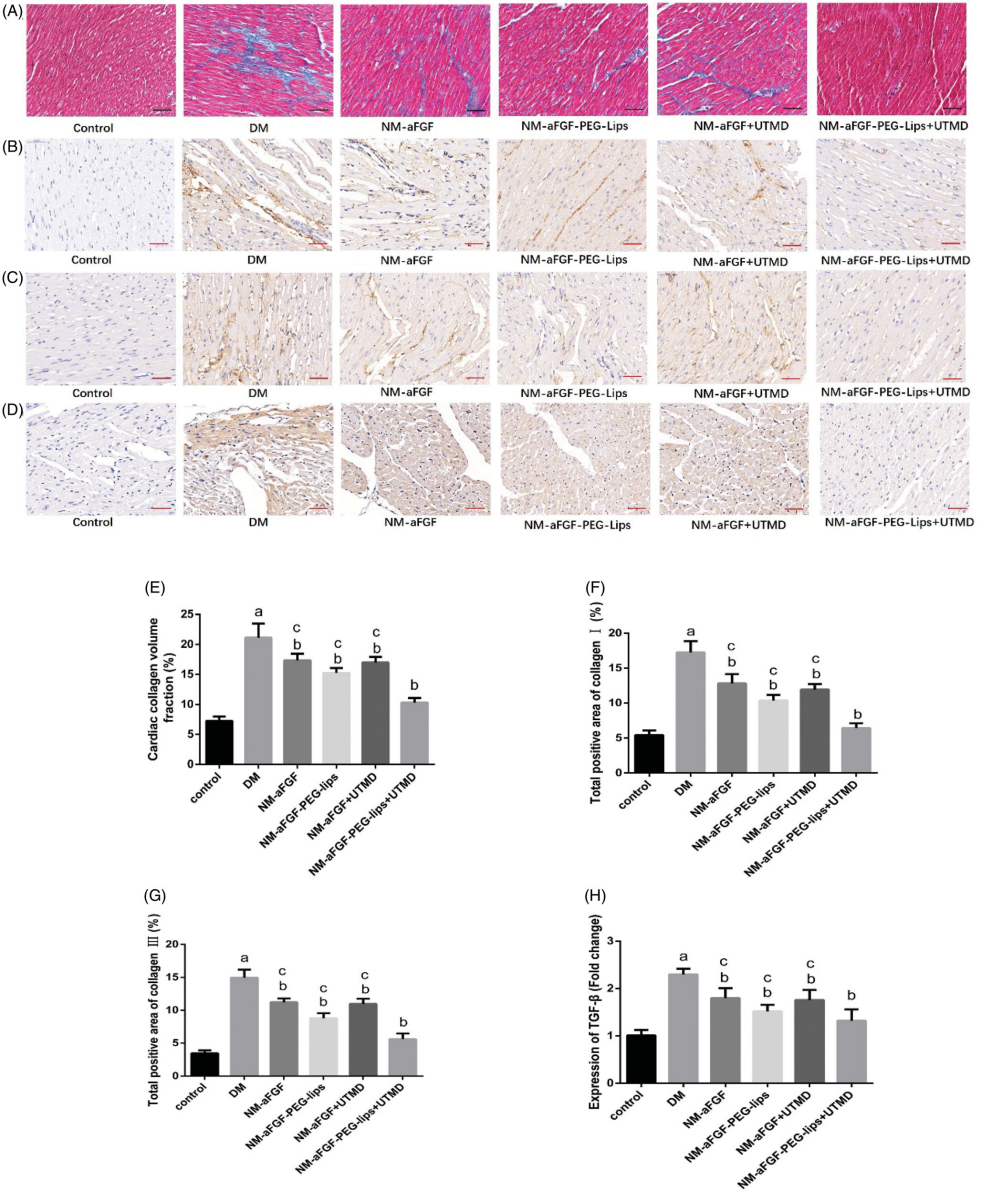
Effects of NM-aFGF-PEG-lips combine with UTMD on cardiac interstitial fibrosis of diabetic rats. (A–D) Representative images of Masson staining and immunohistochemistry staining with collagen I, collagen III and TGF-β1 (400 × ,bar = 50μm). (E) Quantitative analysis of collagen volume fraction (CVF) of rats. *N* = 10 per group. (F–H) Quantitative analyses of the expression of collagen I, collagen III and TGF-β1, respectively. *N* = 10 per group. Data are presented as Mean ± SD. ^a^*p* < .05 vs control group; ^b^*p* < .05 vs DM group; ^c^*p* < .05 vs NM-aFGF-PEG-lips + UTMD. NM-aFGF-PEG-lips: non-mitogenic acidic fibroblast growth factor- PEGylated -liposomes; DM: diabetes mellitus; UTMD: ultrasound-targeted MB destruction.

To gain insight into the effect of NM-aFGF-PEG-lips combined with UTMD on cardiac fibrosis, Masson staining was used to detect the levels of collagen deposition. Representative images of Masson staining (collagen in blue) and the quantification of myocardial collagen volume fraction (CVF) are shown in Figure 4(A,E). Compared with the control group, the DM group rat hearts displayed markedly increased collagen content in the cardiomyocyte interstitial areas. The value of CVF was also increased in the DM group compared with that in the control group (*p* < .05). The NM-aFGF administration groups prominently reduced the value of myocardial CVF compared with the DM group. Among the NM-aFGF administration groups, the NM-aFGF-PEG-lips + UTMD group showed the lowest CVF followed by the NM-aFGF-PEG-lips group and NM-aFGF group. We further determined the alleviative effect of the NM-aFGF-PEG-lips + UTMD treatment on cardiac fibrosis by detecting collagen I, collagen III and TGF-β1 deposition through immunohistochemical staining (Figure 4(B–D)). The quantitative analyses of the expression of collagen I, collagen III and TGF-β1 (Figure 4(F–H)) showed a similar trend consistent with Masson staining.

### Effects of NM-aFGF-PEG-lips combined with UTMD on oxidative stress in cardiac tissues

**Figure 5.**
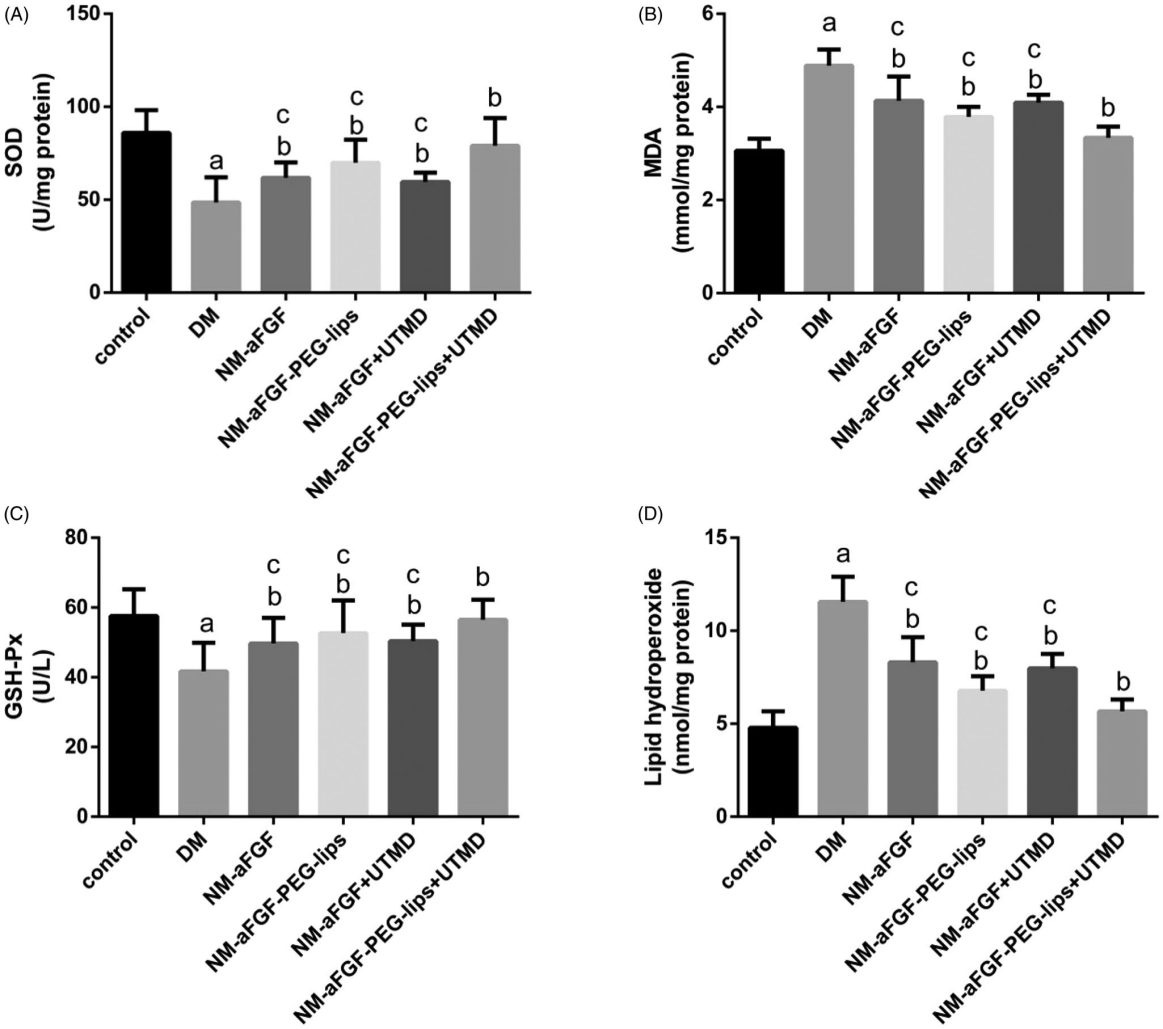
Effect of NM-aFGF-PEG-lips combine with UTMD on antioxidative stress in cardiac tissues. (A) SOD activity; (B) MDA activity; (C) GSH-Px activity and (D) Lipid hydroperoxide contents in the myocardial tissues. *N* = 10 per group. Data are presented as Mean ± SD. ^a^*p* < .05 vs control group; ^b^*p* < .05 vs DM group; ^c^*p* < .05 vs NM-aFGF-PEG-lips + UTMD. NM-aFGF-PEG-lips, non-mitogenic acidic fibroblast growth factor- PEGylated -liposomes; DM: diabetes mellitus; UTMD: ultrasound-targeted MB destruction; SOD: Superoxide dismutase; MDA: malondialdehyde; GSH-Px: glutathione peroxidase.

The antioxidant capacity of NM-aFGF was determined by two pivotal enzymes in scavenging oxygen radicals, SOD and GSH-Px together with the levels of MDA and lipid hydroperoxide. As shown in Figure 5(A–D), the lipid hydroperoxide concentration and MDA level in the DM group were significantly higher than in the control group (*p* < .05), whereas the enzymatic activities of SOD and GSH-Px were dramatically decreased in the DM group compared with the control group (*p* < .05). These changes were all observably reversed by NM-aFGF treatments compared with the DM group (*p* < .05). In all NM-aFGF treatment groups, the therapeutic effect of NM-aFGF-PEG-lips + UTMD was the best.

### Effects of NM-aFGF-PEG-lips combined with UTMD on the protein expression levels of FGF1 and AKT/GSK-3β/Nrff-2-related signaling pathways of oxidative stress in cardiac tissues

**Figure 6.**
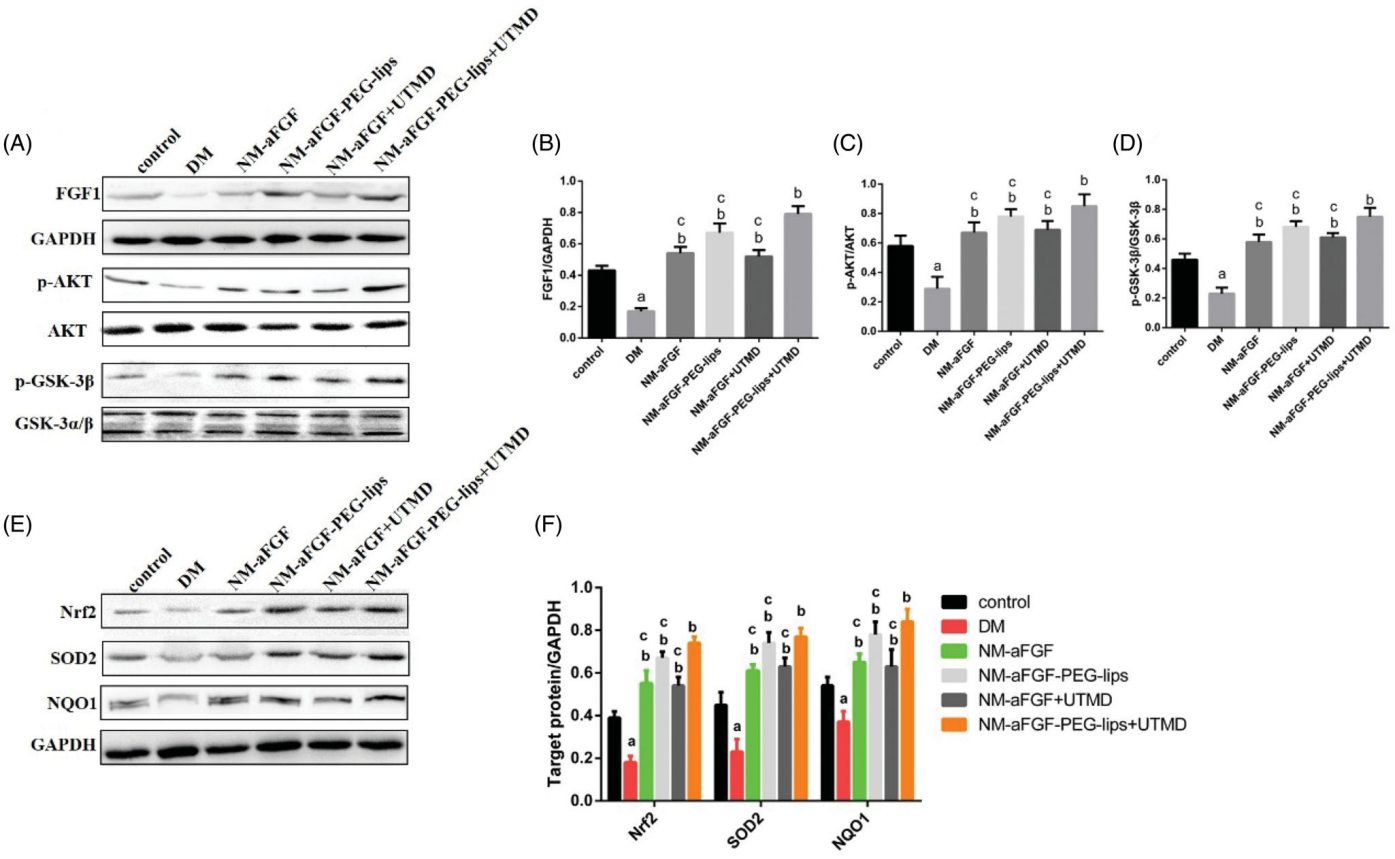
Effects of NM-aFGF-PEG-lips combine with UTMD on the protein expression levels of FGF1 and AKT-related signaling pathways of oxidative stress in cardiac tissues. (A) Expression of FGF1, p-AKT, AKT, p-GSK-3βand GSK-3α/βin cardiac tissues as measured by western blot analyses. (B–D) The quantification data of western blot for FGF1, p-AKT/AKT and p-GSK-3β/GSK-3α/β. (E) Expression of Nrf2, SOD2 and NQO1 in cardiac tissues as detected by western blot analyses. (F) The quantification data of western blot for Nrf2, SOD2 and NQO1. N = 10 per group. Data are presented as Mean ± SD. ^a^*p* < .05 vs control group; ^b^*p* < .05 vs DM group; ^c^*p* < .05 vs NM-aFGF-PEG-lips + UTMD. NM-aFGF-PEG-lips: non-mitogenic acidic fibroblast growth factor- PEGylated -liposomes; DM: diabetes mellitus; UTMD: ultrasound-targeted MB destruction; GAPDH: glyceraldehyde 3-phosphate dehydrogenase.

The protein expression level of FGF1 was detected by Western blot analysis to evaluate the delivery efficiency of NM-aFGF-PEG-lips combined with UTMD. As shown in Figure 6(A–B), the protein expression level of FGF1 was decreased significantly in the DM group compared with the control group (*p* < .05). After treatment with different forms of NM-aFGF, the protein levels of FGF1 were increased significantly compared with the DM group. Furthermore, there was a significant difference in FGF1 protein level between the NM-aFGF-PEG-lips + UTMD group and the other NM-aFGF treatment groups (*p* < .05).

To further elucidate the regulatory mechanism of NM-aFGF pertaining to oxidative stress, we detected the protein levels of the AKT/GSK-3β-related signaling pathways (Figure 6(C–F)). We found that the phosphorylation levels of AKT (p-AKT) and GSK-3β (p-GSK-3β) and the protein expression levels of Nrf2, SOD2, and NQO1 in the DM group were strongly downregulated compared with the control group (*p* < .05). We also found that NM-aFGF upregulated the protein expression levels of p-AKT, p-GSK-3β, Nrf2, SOD2, and NQO1 in diabetic rats with different forms of NM-aFGF treatments compared with the DM group (*p* < .05). Among the NM-aFGF treatment groups, the rats in the NM-aFGF-PEG-lips + UTMD group revealed the highest level of p-AKT, p- GSK-3β, Nrf2, SOD2, and NQO1 protein expression (*p* < .05).

### Effect of NM-aFGF-PEG-lips combined with UTMD on myocardial apoptosis induced by DM

**Figure 7.**
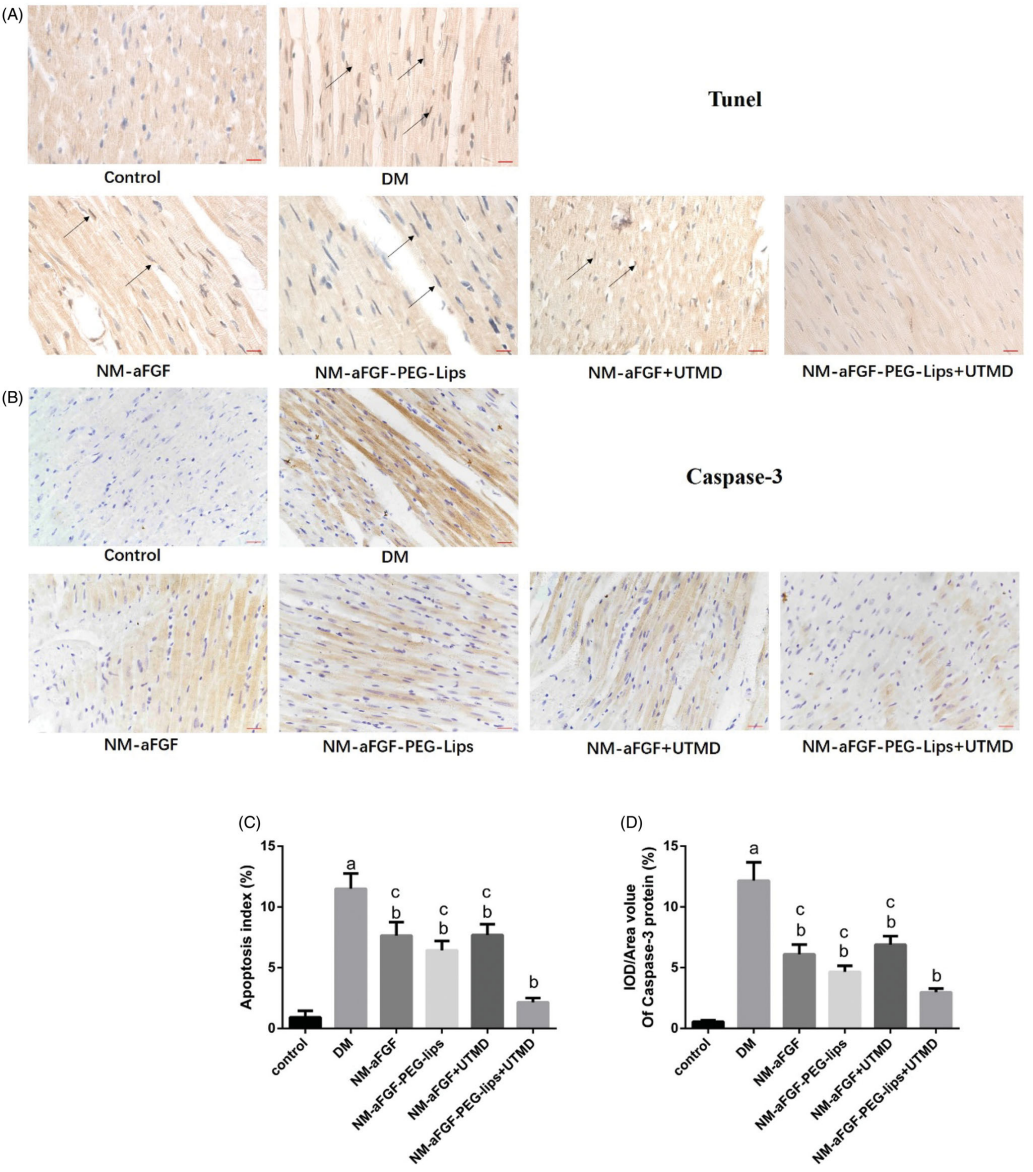
Effect of NM-aFGF-PEG-lips combine with UTMD on myocardial apoptosis induced by DM. (A) Representative images of TUNEL staining. Arrows indicate the nucleus of TUNEL positive cells (400 × ,bar = 50μm). (B) Representative images of immunohistochemistry staining with cleaved caspase-3 (400 × ,bar = 50μm). (C) Quantitative analyses of apoptosis index in myocardial tissues. (D) Quantitative analyses of the expression of cleaved caspase-3 protein in myocardial tissues. *N* = 10 per group. Data are presented as Mean ± SD. ^a^*p* < .05 vs control group; ^b^*p* < .05 vs DM group; ^c^*p* < .05 vs NM-aFGF-PEG-lips + UTMD. NM-aFGF-PEG-lips: non-mitogenic acidic fibroblast growth factor- PEGylated -liposomes; DM: diabetes mellitus; UTMD: ultrasound-targeted MB destruction.

TUNEL staining was performed to evaluate the effect of NM-aFGF-PEG-lips combined with UTMD on DM-induced cardiomyocyte apoptosis. The apoptotic cells were those with brown staining in the nucleus. As shown in Figure 7(A and D), the apoptosis index was significantly increased in the DM group compared with the control group (*p* < .05), and different forms of NM-aFGF interventions significantly decreased the apoptosis index compared with that in the DM group (*p* < .05). Among the NM-aFGF treatment groups, the NM-aFGF-PEG-lips + UTMD group exhibited the lowest apoptosis index compared with the other treatment (*p* < .05). We further detected the expression of apoptosis-related protein cleaved caspase-3 by immunohistochemical staining (Figure 7(B,C)), which showed a similar trend consistent with TUNEL staining.

## Discussion

DCM is a serious and fatal complication of diabetes with a population prevalence of 1.1% (Dandamudi et al., 2014). Evidence suggests that oxidative stress-induced cardiac dysfunction is a key factor in the development of DCM (Xiang et al., 2020). In the present study, we used a structure-based aFGF mutant (NM-aFGF) as a model drug. Through the structural modification of the native aFGF, NM-aFGF retains myocardial protective effects such as antioxidant stress activity and abrogates the oncogenicity of aFGF. As a kind of protein drug, the major drawback of *in vivo* application of NM-aFGF is its rapid clearance from blood due to poor stability. Using nanoliposomes to encapsulate macromolecular protein drugs is one of the most promising and extensively studied strategies for improving the pharmacokinetic behavior of therapeutic drugs by increasing their stability in the blood (Shavi et al., 2016). In this study, we used PEGylated phospholipid (DSPE-PEG 2000) as the membrane material to prepare a novel NM-aFGF-PEG-nanoliposomes by using water-in-water emulsification technology combined with the secondary freeze-drying method. Compared with conventional nanoliposomes, PEG-nanoliposomes can avoid the uptake of mononuclear phagocytic system (MPS) and prolong their circulation time in the blood (Sujitha et al., 2020). Through the investigation of the properties of NM-aFGF-PEG-lips, NM-aFGF-loaded PEG-nanoliposomes were observed to have a round shape with an encapsulation efficiency as high as 87.9 ± 2.1%, which indicates that the novel PEG-nanoliposome can efficiently encapsulate NMFGF1. After different forms of NMFGF1 intervention, the content of FGF1 in myocardial tissue was significantly higher than that in the DCM group and normal control group.

As a novel physiochemical targeted delivery technology, UTMD can temporarily increase the permeability of the cell membrane to increase the uptake of drugs or their carriers in target cells, achieving the target delivery of drug or drug carriers (Forbes et al., 2011). Moreover, ultrasound can also increase the release of drugs from their carriers in target sites, which further accomplishes the target treatment of drugs (Jing et al., 2011). In this study, Western blot analysis was used to evaluate the effectiveness of targeted delivery of NM-aFGF into diabetic myocardial tissues by NM-aFGF-PEG-lips combined with the UTMD technique. Due to the lack of a specific NM-aFGF monoclonal antibody and because NM-aFGF can bind to aFGF monoclonal antibody (Wu et al., 2005), aFGF monoclonal antibody was selected for the Western blotting assay to detect the aFGF content in myocardial tissues (including NM-aFGF and aFGF). The results showed that the endogenous aFGF content in the hearts of DM rats was significantly decreased compared with the control group. However, after different forms of NM-aFGF interventions, the aFGF content in myocardial tissues weas significantly higher than that in the DM group and normal control group. This was partly due to the improvement of cardiac function in DM rats after different forms of NM-aFGF1 interventions, which led to an increase in endogenous aFGF levels in the heart. Another important reason was that exogenous NM-aFGF was given to increase the NM-aFGF levels in the myocardium. In addition, compared with other forms of NM-aFGF intervention groups as well as the DM group and control group, the aFGF content in the NM-aFGF-PEG-lips + UTMD group was significantly increased (*p* < .05). These results indicated that the combination of NM-aFGF-PEG-lips and UTMD could achieve cardiac-targeted delivery of NM-aFGF and significantly increase the levels of NM-aFGF in rat myocardium.

STZ could induce insulin-dependent diabetes by damaging the DNA of pancreatic islet β cells (Radenković et al., 2016). Consistent with previous studies, the type I DM rat model has been well established for research by intraperitoneal injection of STZ, and it would further develop DCM (Guo et al., 2015; Wang et al., 2017). After STZ injection, the rats in our experiment displayed obvious diabetic signs such as hyperglycemia, polyphagia, polydipsia and weight loss. DCM is a typical cardiovascular system complication mediated by diabetes (Diao et al., 2011) and characterized by myocardial left ventricular dysfunction, cardiomyocyte injury, and apoptosis (Zhang et al., 2016). Consistent with those characteristics, we found that the rats in the DM group demonstrated a significant deterioration in LVEF and LVFS at 16 weeks following STZ induction and verification of DM. These results indicate that the cardiac functions were decreased in the DM group. Multiple disorganized myocardial fibers, nuclear vacuolization, more collapse and myofibre vacuolization in the DM group observed by HE staining and TEM further confirmed that rats developed typical DCM after STZ injection. However, compared with the DM group, the cardiac systolic function was significantly improved, and the pathological changes in cardiac myocytes were reversed toward normal conditions in different forms of the NM-aFGF treatments, especially in the NM-aFGF-PEG-lips + UTMD group. Meanwhile, we also found no significant difference in the blood glucose and body weight between the DM group and NM-aFGF intervention groups after 16 weeks of DM. These results indicated that NM-aFGF-PEG-lips combined with UTMD could improve cardiac function and reverse the pathological changes of cardiomyocytes without affecting the blood sugar and body weight of DM rats.

As an important contributor to the development of cardiac dysfunction in DCM, myocardial fibrosis has been found in both type I and type II diabetes (Adeghate, 2004; Tschöpe et al., 2004). The extracellular matrix (ECM) comprises collagen, laminin, elastin and fibronectin (Daniels et al., 2009) abnormally elevated ECM deposition, especially collagen deposition, results in myocardial fibrosis (Huynh et al., 2014). TGF-β1 has been reported to play an important role in regulating collagen generation in DM rats by inducing the differentiation of fibroblasts into myofibroblasts, which have higher collagen production activity than fibroblasts (Wang et al., 2014). In this study, Masson staining was used to measure the collagen volume fraction (CVF), and immunohistochemical staining was also used to detect the expression of collagen I, collagen III and TGF-β1. Our data showed obviously increased CVF and upregulation of collagen I, collagen III and TGF-β11 in rats of the DM group. However, the increased CVF and upregulation of cardiomyocyte fibrosis-associated proteins were significantly attenuated by the treatment of different forms NM-aFGF, especially in the form of NM-aFGF-PEG-lips combined with UTMD. These results indicated that NM-aFGF-PEG-lips + UTMD could efficiently deliver NM-aFGF into cardiac tissues of diabetic rats; furthermore, NM-aFGF had a potentially protective effect on the myocardium against fibrosis by inhibiting TGF-β1-mediated collagen production and deposition.

Cardiomyocyte apoptosis is another key process in DCM. Many studies have found increased cardiomyocyte apoptosis in both diabetic patients and diabetic rat models . The extent of cardiomyocyte apoptosis parallels the severity of the DCM and its stage of evolution (Engel et al., 2004). Caspase-3 is an important factor in regulating apoptosis. Cardiomyocyte apoptosis has been reported to be enhanced by DM-induced activation of caspase-3 pathways (Abdel-Hamid & Firgany Ael, 2015). Caspase-3 is an inactive precursor, and it is activated by converting to cleaved caspase-3 in apoptotic cells (Zidar et al., 2006). In this study, we used TUNEL staining and immunohistochemical staining to investigate the extent of cardiomyocyte apoptosis and the expression of cleaved caspase-3. Our results showed an increased apoptosis index and upregulation cleaved caspase-3 expression in the DM group. However, the apoptosis index and expression of cleaved caspase-3 were significantly decreased in different forms of NM-aFGF intervention groups, especially in the NM-aFGF-PEG-lips + UTMD group. The data indicated NM-aFGF can be efficiently delivered into the cardiac tissues of diabetic rats by PEG-nanoliposomes combined with UTMD, which can play a role in attenuating cardiomyocyte apoptosis caused by DM.

Oxidative stress is known to be associated with the development of DCM. Evidence suggests that oxidative stress is increased in response to hyperglycemia in the vascular tissues of patients with DM, leading to impaired cardiac structure and function in the left ventricle of diabetic rats (Kramer & Zinman, 2016). It is well known that SOD and GSH-px are important enzymes that scavenge reactive oxygen species (ROS) (Pan et al., 2014). MDA is the end-product of lipid peroxidation reaction between ROS and unsaturated fatty acids on the cell membrane, and its content can indirectly reflect the degree of oxidative stress (Malinska et al., 2010). In our experiment, the lipid hydroperoxide and MDA contents as well as the SOD and GSH-px activities were detected to evaluate the level of oxidative stress in cardiac tissue. The results showed a significant increase in oxidative stress in rats of the DM group compared with the control group. However, the oxidative stress was clearly attenuated in the NM-aFGF-PEG-lips + UTMD group compared with in the DCM group and other NM-aFGF treatment groups. These data indicate that NM-aFGF-PEG-lips combined with UTMD can effectively deliver NM-aFGF to myocardial tissues. The NM-aFGF further exerted an effect of attenuating oxidative stress in STZ-induced diabetic rats as illustrated by the decrease in MDA and lipid hydroperoxide and increased SOD and GSH-px activity.

**Figure 8.**
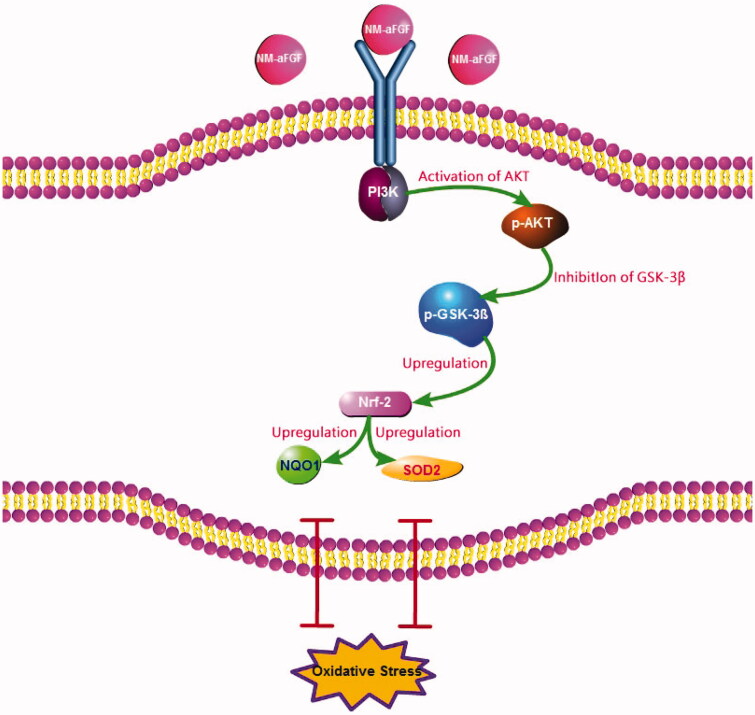
Molecular mechanism of the NM-aFGF mediated inhibition of oxidative stress in rats hearts challenged with DCM. NM-aFGF: non-mitogenic acidic fibroblast growth factor; DCM: diabetic cardiomyopathy.

To further elucidate the regulatory mechanism of NM-aFGF pertaining to oxidative stress, we revisited the Western blotting results. As a downstream target of PI3K, AKT is known as protein kinase B, which is activated by phosphorylation at Ser 308 and Thr 473 (Dhanasekaran et al., 2008). AKT has been reported to have a clearly defined role in the regulation of cardiovascular functions, including cardiac growth and contractile function (Katare et al., 2010). The activity of phosphorylated AKT (p-AKT) is impaired in DCM, leading to myocardial injury and DCM progression (Bilim et al., 2008). Moreover, increased AKT signaling reduces cellular oxidative stress by high blood glucose (Westermann et al., 2007). GSK-3β1, a major substrate of AKT, not only has central functions in glycogen metabolism and insulin resistance (Wu et al., 2014) but also plays a pivotal role in Nrf2 deactivation and the initiation of oxidative injury (Xin et al., 2018). The GSK-3β1 activity was blocked by the phosphorylation at Ser 9 (p-GSK-3β1) after AKT activation. As a downstream target of GSK-3β1, Nrf2 is regarded as important in the cell to protect against oxidative stress by regulating the expression of its downstream proteins, such as NOQ1 and SOD2 (Kobayashi & Yamamoto, 2006). In the present study, we found that the NM-aFGF-lips + UTMD group showed the highest expression of p-AKT, p-GSK3β1, Nrf-2, SOD2, and NOQ1 compared with the DM group, control group and other forms of NM-aFGF-treated groups. Consistent with the results from oxidative stress, these data showed that the combination of NM-aFGF-PEG-lips and UTMD can effectively deliver NM-aFGF to myocardial tissues. NM-aFGF could further activate the AKT/GSK-3β1/Nrf-2 signaling pathway by activating AKT, inhibiting GSK-3β1 as well as upregulating Nrf2, NOQ1 and SOD2 (Figure 8), ultimately exerting myocardium protective effects against oxidative stress in DM rats.

In conclusion, the present study confirmed that using PEGylated nanoliposomes combined with UTMD can effectively deliver NM-aFGF to the cardiac tissue of diabetic rats. NM-aFGF can then inhibit myocardial oxidative stress damage due to DM by activating the AKT/GSK/Nrf-2 signaling pathway, thus improving myocardial structural and functional lesions in diabetic rats. There results provide new guidance for the clinical treatment of DCM.

## References

[CIT0001] Abdel-Hamid AA, Firgany Ael D. (2015). Atorvastatin alleviates experimental diabetic cardiomyopathy by suppressing apoptosis and oxidative stress. J Mol Hist 46:337–45. 10.1007/s10735-015-9625-4 26041576

[CIT0002] Adeghate E. (2004). Molecular and cellular basis of the aetiology and management of diabetic cardiomyopathy: a short review. Mol Cell Biochem 261:187–91. 15362503 10.1023/b:mcbi.0000028755.86521.11

[CIT0003] Ahmadi F, McLoughlin IV, Chauhan S, et al. (2012). Bio-effects and safety of low-intensity, low-frequency ultrasonic exposure. Prog Biophys Mol Biol 108:119–38. 22402278 10.1016/j.pbiomolbio.2012.01.004

[CIT0004] Aisha AF, Majid AM, Ismail Z. (2014). Preparation and characterization of nano liposomes of Orthosiphon stamineus ethanolic extract in soybean phospholipids. BMC Biotechnol 14:23. 24674107 10.1186/1472-6750-14-23 PMC3994274

[CIT0005] Allen TM, Cullis PR. (2013). Liposomal drug delivery systems: from concept to clinical applications. Adv Drug Deliv Rev 65:36–48. 23036225 10.1016/j.addr.2012.09.037

[CIT0006] Bilim O, Takeishi Y, Kitahara T, et al. (2008). Diacylglycerol kinase zeta inhibits myocardial atrophy and restores cardiac dysfunction in streptozotocin-induced diabetes mellitus. Cardiovasc Diabetol 7:2. 18241357 10.1186/1475-2840-7-2 PMC2265681

[CIT0007] Bozzuto G, Molinari A. (2015). Liposomes as nanomedical devices. Int J Nanomedicine 10:975–99. 25678787 10.2147/IJN.S68861 PMC4324542

[CIT0008] Chen M, Bao L, Zhao M, et al. (2020). Progress in research on the role of FGF in the formation and treatment of corneal neovascularization. Front Pharmacol 11:111. 32158390 10.3389/fphar.2020.00111 PMC7052042

[CIT0009] Chen W, Yu M, Wang Y, et al. (2009). Non-mitogenic human acidic fibroblast growth factor reduces retinal degeneration induced by sodium iodate. J Ocul Pharmacol Ther 25:315–20. 19650706 10.1089/jop.2009.0015

[CIT0010] Cui H, Zhu Q, Xie Q, et al. (2020). Low intensity ultrasound targeted microbubble destruction assists MSCs delivery and improves neural function in brain ischaemic rats. J Drug Target 28:320–9. 31429596 10.1080/1061186X.2019.1656724

[CIT0011] Dandamudi S, Slusser J, Mahoney DW, et al. (2014). The prevalence of diabetic cardiomyopathy: a population-based study in Olmsted County, Minnesota. J Card Fail 20:304–9. 24576788 10.1016/j.cardfail.2014.02.007 PMC4076144

[CIT0012] Daniels A, van Bilsen M, Goldschmeding R, et al. (2009). Connective tissue growth factor and cardiac fibrosis. Acta Physiol (Oxf) 195:321–38. 19040711 10.1111/j.1748-1716.2008.01936.x

[CIT0013] Dhanasekaran A, Gruenloh SK, Buonaccorsi JN, et al. (2008). Multiple antiapoptotic targets of the PI3K/Akt survival pathway are activated by epoxyeicosatrienoic acids to protect cardiomyocytes from hypoxia/anoxia. Am J Physiol Heart Circ Physiol 294:724–35. 10.1152/ajpheart.00979.2007 PMC2443685 18055514

[CIT0014] Engel D, Peshock R, Armstong RC, et al. (2004). Cardiac myocyte apoptosis provokes adverse cardiac remodeling in transgenic mice with targeted TNF overexpression. Am J Physiol Heart Circ Physiol 287:1303–11. 10.1152/ajpheart.00053.2004 15317679

[CIT0015] Forbes MM, Steinberg RL, O'Brien WD. Jr. (2011). Frequency-dependent evaluation of the role of definity in producing sonoporation of Chinese hamster ovary cells. J Ultrasound Med 30:61–9. 21193706 10.7863/jum.2011.30.1.61 PMC3069854

[CIT0016] Galderisi M. (2006). Diastolic dysfunction and diabetic cardiomyopathy: evaluation by Doppler echocardiography. J Am Coll Cardiol 48:1548–51. 17045886 10.1016/j.jacc.2006.07.033

[CIT0017] Giacco F, Brownlee M. (2010). Oxidative stress and diabetic complications. Circ Res 107:1058–70. 21030723 10.1161/CIRCRESAHA.110.223545 PMC2996922

[CIT0018] Guan Y, Cai B, Liu Z, et al. (2016). The formation of aberrant collateral vessels during coronary arteriogenesis in dog heart. Cells Tissues Organs (Print) 201:118–29. 10.1159/000442381 26796132

[CIT0019] Guo R, Liu W, Liu B, et al. (2015). SIRT1 suppresses cardiomyocyte apoptosis in diabetic cardiomyopathy: an insight into endoplasmic reticulum stress response mechanism. Int J Cardiol 191:36–45. 25965594 10.1016/j.ijcard.2015.04.245

[CIT0020] Diao X, Shen E, Wang X, et al. (2011). Differentially expressed microRNAs and their target genes in the hearts of streptozotocin-induced diabetic mice. Mol Med Rep 4:633–40. 21584493 10.3892/mmr.2011.489

[CIT0021] Huang C, Li C, Muhemaitia P. (2019). Impediment of selenite-induced cataract in rats by combinatorial drug laden liposomal preparation. Libyan J Med 14:1548252. 30460877 10.1080/19932820.2018.1548252 PMC6249608

[CIT0023] Huynh K, Bernardo BC, McMullen JR, et al. (2014). Diabetic cardiomyopathy: mechanisms and new treatment strategies targeting antioxidant signaling pathways. Pharmacol Ther 142:375–415. 24462787 10.1016/j.pharmthera.2014.01.003

[CIT0024] Jing D, Shi QS, Sun Y, et al. (2011). Enhanced delivery of monomethoxypoly(ethylene Glycol)-Poly(lactic-Co-Glycolic Acid)-Poly L-lysine nanoparticles loading platelet-derived growth factor bb small interfering rna by ultrasound and/or microbubbles to rat retinal pigment epithelium cells. J Gene Med 13:312–23. 21674734 10.1002/jgm.1574

[CIT0025] Katare RG, Caporali A, Oikawa A, et al. (2010). Vitamin B1 analog benfotiamine prevents diabetes-induced diastolic dysfunction and heart failure through Akt/Pim-1-mediated survival pathway. Circ Heart Fail 3:294–305. 20107192 10.1161/CIRCHEARTFAILURE.109.903450 PMC2865995

[CIT0026] Kobayashi M, Yamamoto M. (2006). Nrf2-Keap1 regulation of cellular defense mechanisms against electrophiles and reactive oxygen species. Adv Enzyme Regul 46:113–40. 16887173 10.1016/j.advenzreg.2006.01.007

[CIT0027] Kramer CK, Zinman B. (2016). Sodium-glucose co-transporter-2 (SGLT-2) inhibitors in patients with type 2 diabetes mellitus: the road ahead. Eur Heart J 37:3201–2. 27153862 10.1093/eurheartj/ehw158

[CIT0028] Lentacker I, De Cock I, Deckers R, et al. (2014). Understanding ultrasound induced sonoporation: definitions and underlying mechanisms. Adv Drug Deliv Rev 72:49–64. 24270006 10.1016/j.addr.2013.11.008

[CIT0029] Lu CT, Zhao YZ, Wu Y, et al. (2011). Experiment on enhancing antitumor effect of intravenous epirubicin hydrochloride by acoustic cavitation in situ combined with phospholipid-based microbubbles. Cancer Chemother Pharmacol 68:343–8. 20978763 10.1007/s00280-010-1489-4

[CIT0030] Malinska D, Kulawiak B, Kudin AP, et al. (2010). Complex III-dependent superoxide production of brain mitochondria contributes to seizure-related ROS formation. Biochim Biophys Acta 1797:1163–70. 20211146 10.1016/j.bbabio.2010.03.001

[CIT0031] Miller DL, Dou C. (2009). Induction of apoptosis in sonoporation and ultrasonic gene transfer. Ultrasound Med Biol 35:144–54. 18723272 10.1016/j.ultrasmedbio.2008.06.007 PMC2642595

[CIT0032] Pan LH, Li XF, Wang MN, et al. (2014). Comparison of hypoglycemic and antioxidative effects of polysaccharides from four different Dendrobium species. Int J Biol Macromol 64:420–7. 24370475 10.1016/j.ijbiomac.2013.12.024

[CIT0033] Panje CM, Wang DS, Pysz MA, et al. (2012). Ultrasound-mediated gene delivery with cationic versus neutral microbubbles: effect of DNA and microbubble dose on *in vivo* transfection efficiency. Theranostics 2:1078–91. 23227124 10.7150/thno.4240 PMC3516840

[CIT0034] Pappachan JM, Varughese GI, Sriraman R, et al. (2013). Diabetic cardiomyopathy: pathophysiology, diagnostic evaluation and management. World J Diabetes 4:177–89. 24147202 10.4239/wjd.v4.i5.177 PMC3797883

[CIT0036] Pili R, Chang J, Muhlhauser J, et al. (1997). Adenovirus-mediated gene transfer of fibroblast growth factor-1: angiogenesis and tumorigenicity in nude mice. Int J Cancer 73:258–63. 9335452 10.1002/(sici)1097-0215(19971009)73:2<258::aid-ijc16>3.0.co;2-b

[CIT0037] Radenković M, Stojanović M, Prostran M. (2016). Experimental diabetes induced by alloxan and streptozotocin: the current state of the art. J Pharmacol Toxicol Methods 78:13–31. 26596652 10.1016/j.vascn.2015.11.004

[CIT0038] Scarlett JM, Rojas JM, Matsen ME, et al. (2016). Central injection of fibroblast growth factor 1 induces sustained remission of diabetic hyperglycemia in rodents. Nat Med 22:800–6. 27213816 10.1038/nm.4101 PMC4938755

[CIT0039] Shavi GV, Sreenivasa Reddy M, Raghavendra R, et al. (2016). PEGylated liposomes of anastrozole for long-term treatment of breast cancer: *in vitro* and *in vivo* evaluation. J Liposome Res 26:19. 10.3109/08982104.2015.1029493 25853340

[CIT0040] Sheng WS, Xu HL, Zheng L, et al. (2018). Intrarenal delivery of bFGF-loaded liposome under guiding of ultrasound-targeted microbubble destruction prevent diabetic nephropathy through inhibition of inflammation. Artif Cells Nanomed Biotechnol 46:373–85. 29653493 10.1080/21691401.2018.1457538

[CIT0042] Sujitha S, Dinesh P, Rasool M. (2020). Berberine encapsulated PEG-coated liposomes attenuate Wnt1/β-catenin signaling in rheumatoid arthritis via miR-23a activation. Eur J Pharm Biopharm 149:170–91. 32068029 10.1016/j.ejpb.2020.02.007

[CIT0043] Tian XQ, Ni XW, Xu HL, et al. (2017). Prevention of doxorubicin-induced cardiomyopathy using targeted MaFGF mediated by nanoparticles combined with ultrasound-targeted MB destruction. Int J Nanomedicine 12:7103–19. 29026304 10.2147/IJN.S145799 PMC5627735

[CIT0044] Tschöpe C, Walther T, Königer J, et al. (2004). Prevention of cardiac fibrosis and left ventricular dysfunction in diabetic cardiomyopathy in rats by transgenic expression of the human tissue kallikrein gene. Faseb J 18:828–35. 15117887 10.1096/fj.03-0736com

[CIT0045] Wang WK, Wang B, Lu QH, et al. (2014). Inhibition of high-mobility group box 1 improves myocardial fibrosis and dysfunction in diabetic cardiomyopathy. Int J Cardiol 172:202–12. 24485636 10.1016/j.ijcard.2014.01.011

[CIT0046] Wang XM, Wang YC, Liu XJ, et al. (2017). BRD7 mediates hyperglycaemia-induced myocardial apoptosis via endoplasmic reticulum stress signalling pathway. J Cell Mol Med 21:1094–105. 27957794 10.1111/jcmm.13041 PMC5431142

[CIT0047] Westermann D, Van Linthout S, Dhayat S, et al. (2007). Cardioprotective and anti-inflammatory effects of interleukin converting enzyme inhibition in experimental diabetic cardiomyopathy. Diabetes 56:1834–41. 17473225 10.2337/db06-1662

[CIT0048] Wu X, Su Z, Li X, et al. (2005). High-level expression and purification of a nonmitogenic form of human acidic fibroblast growth factor in Escherichia coli. Protein Expr Purif 42:7–11. 15882952 10.1016/j.pep.2004.07.021

[CIT0049] Wu Z, Chen Q, Ke D, et al. (2014). Emodin protects against diabetic cardiomyopathy by regulating the AKT/GSK-3β signaling pathway in the rat model. Molecules 19:14782–93. 25232702 10.3390/molecules190914782 PMC6271268

[CIT0050] Xiang L, Zhang Q, Chi C, et al. (2020). Curcumin analog A13 alleviates oxidative stress by activating Nrf2/ARE pathway and ameliorates fibrosis in the myocardium of high-fat-diet and streptozotocin-induced diabetic rats. Diabetol Metab Syndr 12:1–8. 31921358 10.1186/s13098-019-0485-z PMC6947902

[CIT0051] Xiao J, Lv Y, Lin S, et al. (2010). Cardiac protection by basic fibroblast growth factor from ischemia/reperfusion-induced injury in diabetic rats. Biol Pharm Bull 33:444–9. 20190407 10.1248/bpb.33.444

[CIT0052] Xin Y, Bai Y, Jiang X, et al. (2018). Sulforaphane prevents angiotensin II-induced cardiomyopathy by activation of Nrf2 via stimulating the Akt/GSK-3ß/Fyn pathway. Redox Biol 15:405–17. 29353218 10.1016/j.redox.2017.12.016 PMC5975128

[CIT0053] Yuba E, Harada A, Sakanishi Y, et al. (2013). A liposome-based antigen delivery system using pH-sensitive fusogenic polymers for cancer immunotherapy. Biomaterials 34:3042–52. 23374704 10.1016/j.biomaterials.2012.12.031

[CIT0054] Zhang M, Yu WZ, Shen XT, et al. (2016). Advanced interfere treatment of diabetic cardiomyopathy rats by aFGF-loaded heparin-modified microbubbles and UTMD technique. Cardiovasc Drugs Ther 30:247–61. 26947349 10.1007/s10557-016-6639-4

[CIT0055] Zhang Z, Zhang D, Dou M, et al. (2016). Dendrobium officinale Kimura et Migo attenuates diabetic cardiomyopathy through inhibiting oxidative stress, inflammation and fibrosis in streptozotocin-induced mice. Biomed Pharmacother 84:1350–8. 27802903 10.1016/j.biopha.2016.10.074

[CIT0056] Zhao YZ, Li X, Lu CT, et al. (2014). Gelatin nanostructured lipid carriers-mediated intranasal delivery of basic fibroblast growth factor enhances functional recovery in hemiparkinsonian rats. Nanomedicine 10:755–64. 24200526 10.1016/j.nano.2013.10.009

[CIT0057] Zhao YZ, Zhang M, Wong HL, et al. (2016). Prevent diabetic cardiomyopathy in diabetic rats by combined therapy of aFGF-loaded nanoparticles and ultrasound-targeted microbubble destruction technique. J Control Release 223:11–21. 26712588 10.1016/j.jconrel.2015.12.030

[CIT0058] Zidar N, Dolenc-Strazar Z, Jeruc J, et al. (2006). Immunohistochemical expression of activated caspase-3 in human myocardial infarction. Virchows Arch 448:75–9. 16205944 10.1007/s00428-005-0073-5

